# Supplementary material for: Are carbon emissions trading and green financial instruments synergistic? -Comprehensive quantitative research based on content analysis
Source: PLoS One. 2024 Mar 7;19(3):e0298601. doi: 10.1371/journal.pone.0298601 (PMC10919601; doi:10.1371/journal.pone.0298601)
Supplement: S1 Data — (ZIP) [file pone.0298601.s001.zip › Data/地级市Z_F工作报告.zip!/║■─╧/┬a╡╫╩╨/└┤╫╘╣1⁄2╓┌║┼í╛┬φ┐╦╩2╛▌═°í┐_2015.docx]

各位代表：

    现在，我代表市人民政府，向大会作政府工作报告，请予审查，并请各位政协委员和列席人员提出意见。

    一、2014年工作回顾

    2014年，是极不平凡、极其艰难的一年，是近年来我市困难最多、压力最大的一年。面对经济下行压力增大、结构调整阵痛加剧、企业生产经营困难、民间融资风险显现的严峻形势，在省委、省政府和市委的坚强领导下，在市人大、市政协、娄底军分区及娄底父老乡亲强有力的支持下，全市上下立足娄底实际，始终坚持以民生为本、发展为要，牢牢把握“稳中求进、三量齐升”，坚定不移推进“科学发展、加速转型”，凝心聚力、竭尽全力解决发展与民生中一些现实而又关键的问题，取得了较好成效。这一年，我们科学应对“四期叠加”的深度影响，全力以赴稳增长、促转型，在异常艰难的形势下，努力保持了全市经济“稳中趋缓、稳中有进”的良好态势。这一年，我们卓有成效地推进了以交通为重点的基础设施建设，长韶娄高速、新溆高速、安邵高速涟源段顺利通车，娄衡、益娄高速加速推进，特别是沪昆高铁娄底段正式运行，从根本上结束了我市“外无出路、内无好路”的历史，迎来了千载难逢的高铁新时代。这一年，我们高度重视民生改善，全力以赴化解城区大班额问题，娄底一中城南校区等一批新学校、新校舍交付使用，实现了中心城区义务教育学校班额新生65人以下、老生75人以下的目标，各县（市、区）大班额问题也有明显缓解。这一年，我们全市上下戮力同心，成功举办第十二届省运会、第九届省残运会、第五届湘博会、第九届湖南西部农博会等大型盛会，创建国家卫生城市进入拟命名公示阶段，展示了形象，聚集了人气，提升了影响力。这一年，我们全力争取省委、省政府的重视和银信部门的支持，依法、科学而又务实地处置民间融资集中爆发问题，坚决守住了不发生区域性、系统性金融风险和重大群体性事件两条底线。这一年，我们积极争取国家和省重大政策支持，全市被国家确定为全国循环经济示范市和国家二级物流节点市，并整体纳入湘西地区开发范围和武陵山片区生态文明先行示范区，双峰县纳入了全省特色产业县，冷水江锡矿山区域转型发展纳入了省五大重点支持范围，特别是省政府专门召开常务会议，研究出台《关于支持娄底市资源型城市转型发展实施意见》，为我市加快发展注入了巨大能量。这一年，我们深入开展群众路线教育实践活动，认真贯彻执行中央“八项规定”和省委“九项规定”，严厉整治“四风”问题，党员、干部担当精神和进取意识进一步增强，各级政府公信力、推动力、执行力得到提高。成绩可圈可点，具体报告如下。

（一）经济发展稳中提质。运行总体稳健。全年实现地区生产总值1210.91亿元，增长8.1%；完成公共财政总收入95.3亿元，地方公共财政预算收入58.9亿元。质量有所提升。三次产业比调整为14.6:53.7:31.7。工业结构逐步优化，实现规模以上工业增加值431.12亿元，增长9.5%，轻工业增长24.8%，高于重工业增速17.3个百分点，高新技术产业、高加工度产业分别增长11.0%、16.7%，六大高耗能行业增加值占比下降3.2个百分点。园区建设来势较好，全年新建标准厂房85.5万平方米，省级以上产业园区规模工业增长12.1%，占全市规模工业的38.3%。三产稳中显优，全国中小商贸流通企业服务体系建设试点全面启动，住宿餐饮、批发零售、金融、信息、电子商务、健康养老等服务业发展势头良好。文化旅游融合发展，全年接待游客人次、旅游总收入分别增长22.54%、23.1%。新化县成功列入全国休闲农业与乡村旅游示范县，涟源市成功创建全省旅游强县并获评全省旅游十佳县。消费水平稳步提升，实现社会消费品零售总额381.35亿元，增长12.5%。对外贸易恢复性增长，完成进出口总额16.6亿美元，增长11.5%。农业现代化水平不断提高，全年播种粮食410.31万亩，总产161.45万吨，分别增长0.7%、2.5%。产业化水平不断增强，新增省级农业、林业龙头企业13家，农产品加工企业达到1130家，“三品一标”农业企业达到21家，“三黑一菌”特色产业不断壮大。农业机械化程度进一步提升。涟源市成功创建第三批国家现代农业示范区。后劲不断增强。全年完成固定资产投资938.9亿元，增长19.2%，施工项目1791个，新开工项目1261个，竣工项目1011个，汽车板、湖南海螺二期、天宏不锈钢一期、天客物流、华天大酒店等一批重大项目竣工投产，湘中国际物流园、中国诚通物流园、众一健康城等项目进展良好。

（二）城乡建设取得突破。交通条件极大改善，除高铁、高速建设取得突破外，干线公路改造全面推进，全年新改建干线公路135.6公里、城市道路52.4公里、农村公路632.9公里。融城战略顺利实施，娄底大道加速建设，“两大经济板块”、“两条经济走廊”格局初显雏形，沪昆高铁南站枢纽一体化项目有序推进，万宝新区高铁片区路网基本成型，城市骨架进一步拓展；中心城区内环线、二水厂提质改造等项目建成投入使用，湘阳东街等19条道路提质改造全部完成。冷水江市城东生态城、涟源市城东新区、双峰县城北新区、新化县北塔区建设稳步推进。8个全国重点镇完成污水管网建设46.3公里，21个省级示范镇、中心镇、特色镇建设加速推进。全市城镇化水平比上年提高1.32个百分点，达到42.28%。新农村建设6大示范片区建设顺利推进，3个省级新农村建设示范村、11个美丽乡村示范村建设圆满完成。水府庙湿地公园获批国家湿地公园。城乡环境整治与建设四年行动计划成效明显，完成造林15.6万亩，四水治理和中小河流治理、小型农田水利重点县建设、70座小型水库除险加固完成年度任务，农村垃圾分类减量无害化处理试点成功，农村卫生状况明显好转，全市2706个村达到整治标准，388个人居环境整治村通过省级验收，冷水江整市推进农村环境综合整治进展顺利，双峰县荷叶镇跻身全国文明村镇行列，新化县正龙村荣获“中国最美休闲乡村”称号。

（三）发展活力不断增强。各项改革稳步启动。经济体制改革加快推进，国企改革进一步深化，省直管县体制改革、财税体制、园区管理体制、产权制度和要素市场改革、农村土地承包经营权确权颁证等改革启动实施，万宝新区两型示范建设步伐加快。行政审批清理取得突破，市本级仅保留行政许可项目172项，项目审批提速50%以上。市县政府机构改革稳步推进，市政府机构改革方案已获省批复。工商登记制度改革成效明显，新设企业3616户，新增注册资本173.26亿元。财政涉农资金整合试点有序推进，已整合涉农资金6.6亿元。金融改革进一步深化，双峰农商银行挂牌开业。创新驱动成效明显，已建成国家级检验检测中心、企业技术中心各1个，省级工程技术研究中心3个、检验检测中心2个、企业技术中心9个，获省科技进步一等奖、二等奖各1项，省专利奖3项，冷水江市获得国家科技惠民支持。开放合作不断深化，新增广东省佛山市为友好城市，全年实际利用外资3.25亿美元，增长15.6%，增幅居全省第一；内联引资388.9亿元，增长27.5%。

（四）民生福祉持续改善。全面小康社会总实现程度预计达80.4%。改善民生力度加大。全年民生支出138.07亿元，比上年增加12.68亿元，占公共财政预算支出的67.98%。29个为民办实事项目圆满完成。新增城镇就业人员4.12万人，农村劳动力转移就业5.66万人。城、乡居民人均可支配收入预计分别达到20324元、7835元，增长8.8%、11.0%。投入各类扶贫资金6.5亿元，减少贫困人口9.95万人。全面完成全国水库移民避险解困第一批试点任务，新化模式在全国推广。实施统一的城乡居民养老保险，企业退休职工养老金已连续10年上调。医疗、生育、工伤、失业保险工作稳步推进。城、乡低保救助水平逐步提高，农村低保基本实现应保尽保，农村五保分散和集中供养标准分别提高到每人每年2640元和5500元以上。全市新开工保障性住房37743套、基本建成23082套、竣工8800套、分配入住2347套，改造农村危房6000户，保障性住房工作连续2年位居全省前列。解决农村30万人饮水不安全问题，乡镇全部通上了自来水。价格调控务实有效，“菜篮子”工程成效显著，市场供给价稳量足质好。教育质量稳中有升，全年财政教育支出34.3亿元、义务教育保障资金4.2亿元，建设合格学校159所。公共卫生与医疗服务改革稳步推进，三级医院由1家发展至4家，医疗综合服务能力明显提升。新农合参合率达99.2%，补助农民看病421万人次、金额12.18亿元。文化惠民扎实推进，市文化中心竣工，新化县被评为全国文化先进县，双峰县被评为全省民俗文化县。“单独两孩”政策稳步实施，人口自然增长率控制在7.5‰以内，连续8年被评为全省计划生育工作先进单位。慈善事业健康发展，我市荣获“中国六星级慈善城市”称号。

（五）社会大局和谐稳定。金融风险有效稳控。坚决落实“该稳则稳、该退则退、能扶则扶”的精神，严格按照“党政统揽、属地管理，一线担当、企业主体，分类处置、平稳过渡”的总体思路，全力以赴处置民间融资问题，部分企业通过全力帮扶已步入正常生产经营轨道。成功举办省市金融机构支持娄底实体经济发展政银企合作对接会，签约融资项目182个、金额200.6亿元。通过多措并举，保障了金融市场的稳定繁荣，全市银行机构本外币存款余额1126.1亿元，比年初增加105.8亿元；本外币贷款余额704.2亿元，比年初增加74.3亿元；余额贷存比达62.5%，稳居全省第4位。生产安全持续好转。在全国率先出台《预防安全生产事故责任追究暂行规定》。通过实施“四大三基”行动，对20类违法违规行为进行精准整治，排查治理安全隐患2.8万余处。首次实现工矿商贸类企业未发生较大及以上生产安全事故的目标，取得了连续5年未发生重特大生产安全事故的历史最好成绩。联合强力治超全面启动，“三超”现象得到有效遏制。生态环境逐步改善。节能减排任务圆满完成，再生资源回收体系建设步伐加快。九大环保工程、锡矿山地区环境整治与生态修复示范区建设等项目加快推进。争取国家批准冷水江市发行12亿元湘江流域重金属污染治理专项债券，推进了砷碱渣无害化处理一期工程等一批项目竣工运行。实行最严格的水资源管理制度，湘江保护一号重点工程、“四水三库”保护治理取得阶段性成果，全市地表水水质显著改善，饮用水源水质达标率100%。实施大气污染防治计划，中心城区全年环境空气质量达标率达94.5%。社会治理不断加强。平安娄底建设扎实推进，城区实现天网工程全覆盖,各县市天网工程全面推进，扫黑除恶、打击“两抢一盗”等专项行动成效显著，群体性事件有效遏制。基层组织建设不断加强，第九次村委会换届选举圆满完成。社区标准化试点、社区网格治理全面推进，“三调联动”机制有效实施，领导干部接访下访深入推进，23件省交办积案全部办结，市交办积案到期办结率达85.7%。社会管理综合治理民调位列全省第7位，排名大幅提升。

与此同时，市政府及工作部门自觉执行市人大及其常委会的决定，主动接受人大法律监督与工作监督、政协民主监督，充分吸纳各民主党派、工商联和社会各界的意见、建议。全年共办理代表建议226件，政协委员建议案1件，提案170件。对中心城区限制燃放烟花鞭炮的议案，已制定详细方案并进行公告，今年4月份即将全面实施。成功创建全省双拥模范城，国防后备力量建设有效推进，全民国防教育深入普及，聚焦强军目标抓实战化训练初见成效，涉军维权扎实有效，驻娄解放军、武警、消防、公安和民兵预备役人员履行使命任务能力持续提升，圆满完成各类急难险重任务，为全市经济社会发展与稳定作出了重要贡献。

审计、统计、档案、保密、人防、地震、老龄、外事侨务、科普、民族宗教、地方志等工作得到加强，工会、共青团、妇女儿童、科协、对台、贸促会、红十字、残联、社科联、住房公积金等各项事业取得新进步。

各位代表，过去的一年，全市上下同心协力、同舟共济、同甘共苦，顶住了压力、化解了危机、确保了稳定、推进了发展。这些成绩的取得，是市委总揽全局、坚强领导的结果，是市人大、市政协、娄底军分区有力监督、鼎力支持的结果，是全市父老乡亲和社会各界攻坚克难、团结奋进的结果。在此，我谨代表市人民政府，向各位代表、政协委员，向全市父老乡亲，向广大干部职工、政法干警、驻娄解放军和武警、消防官兵，向各民主党派、工商联、人民团体，向所有关心支持我市发展的各界人士，表示衷心的感谢并致以崇高的敬意！

在总结成绩的同时，我们也清醒地看到，我市经济社会发展中还面临着一些困难和问题：一是增长速度放缓，财税收入等个别指标未实现预期目标，经济增长面临更大压力；二是结构调整阵痛显现，企业生产经营困难，加速转型面临更大压力；三是宏观调控趋紧，增长动力不足，可持续发展面临更大压力；四是各类经济风险逐步显现，资源产业发展中历史欠账仍然较多，维护稳定面临更大压力。与此同时，开放合作意识不强、工作作风不实、发展环境不优等问题仍然突出。对此，我们将高度重视，认真加以解决。

二、2015年工作任务

2015年，既是我国全面深化改革的关键之年、全面推进依法治国的开局之年，也是检验我市“十二五”规划完成情况、六个“四年行动计划”推进成效的收官之年，更是我们面对经济发展新常态，务必坚定信心，把握机遇，应对挑战，进一步抓实转型发展、社会稳定、改革创新的关键之年，做好今年政府的各项工作意义重大而深远。在此关键时期，信心比黄金更重要。我们的信心来自于日益显现的区位优势，全省“一核六轴”空间布局我市占据其二，加上交通路网的完善、重要物流节点城市的定位、重大产业项目的布局，我市由概念上的“几何中心”一跃而成为名副其实的交通枢纽、流通中心；我们的信心来自于日趋完备的产业基础，随着汽车板、湘中国际物流园等一大批战略性新兴产业投产达效，农机、煤机、电子陶瓷等特色产业的提质增效，新的增长点正在不断形成；我们的信心来自于趋于坚实的发展平台，娄底城镇带建设进入省级规划，融城战略加快推进，各类园区快速发展，发展空间进一步拓展；我们的信心来自于坚强有力的政策支持，资源型城市转型、两型示范片区创建、产业特色县建设等方面争取的重大政策支持，将为我市发展注入不竭动力；我们的信心来自于全市人民更加理性务实所形成的强大合力，在应对各类风险和各种矛盾中，我们经受了锤炼和考验，对娄底未来的发展、对我们每个娄底人自身的事业与发展，大家决不会再盲目，而一定会更加理性、更加务实、更有合力。我坚信，只要我们全市上下精诚团结，充满信心，理性认清市情、准确把握机遇、充分激活潜力，就一定能够在困境中化危为机，就一定能够在发展中赢得先机。

2015年政府工作的总体思路是：全面贯彻落实党的十八大和十八届三中、四中全会，中央、省委、市委经济工作会议精神，牢牢把握“发展优先、稳中求进”的工作总基调，全面推进“科学发展、加速转型”，以全面建成小康社会为总揽，主动适应经济发展新常态，积极对接大长沙、主动融入长株潭，进一步提质量增效益、转方式调结构、抓改革促创新、控风险守底线、惠民生保和谐，紧紧围绕“三量齐升”，务实推进“四化两型”，全力打好六个“四年行动计划”攻坚战，奋力开创全市经济社会发展新局面。

2015年全市经济社会发展的主要预期目标是：地区生产总值增长8.5%，其中一、二、三产业增加值分别增长3.5%、9%和9.5%；规模以上工业增加值增长9%以上；固定资产投资增长18%；公共财政总收入、地方公共财政预算收入均增长6%；社会消费品零售总额增长12%；实现进出口总额19.2亿美元；城乡居民收入分别增长9%、11.5%以上；人口自然增长率、城镇登记失业率分别控制在7.5‰、4.3%以内；节能减排完成省定目标。

围绕上述目标，重点抓实六个方面的工作。

（一）进一步抓实企业发展。企业是市场经济的主体，是经济发展的主体，更是税源保障的主体，我们务必强化企业意识，依据法律和政策着力帮扶企业，切实解决企业生产、经营、发展中的实际问题。

着力强化要素保障。多方施策缓解用地压力。用好用活土地政策，实施用地与投资强度、税收贡献“双挂钩”，推动土地节约集约利用。盘活土地存量，充分置换现有土地，积极清理各类闲置用地、低效用地，符合无偿收回条件的应予收回，应该征缴闲置费的坚决征缴。加大征地拆迁工作力度，坚持安置先行，切实维护被征地农民的利益，确保企业项目顺利推进。完善水、电、路、气配套建设，保证企业能源供给。强化人才保障，加快出台政策措施，加强企业家队伍建设，支持企业引进和培养高端创新型人才、高级管理人才、高层次专业技术人才和高技能人才，加大职业培训力度，加强企业用工保障。

着力促进投产达效。对重点企业及项目实行点对点服务，开辟绿色通道，特事特办、急事急办，确保在建企业项目早竣工、早投产、早见效。引导企业科学定位，理性发展，强化内部管理，进一步优化产品结构，巩固传统市场、开拓新兴市场，在充分认识行业形势、自身发展潜力和盈利水平的基础上，合理确定发展方向和规模。出台政策，引导政府采购、本地企业使用本地产品，帮助企业渡过难关。

着力处置金融风险。坚持“党政统揽、属地管理，一线担当、企业主体，分类处置、平稳过渡”总体思路，全力以赴加强民间融资风险处置，鼓励企业生产自救，力争取得实质性突破，坚决杜绝区域性、系统性金融风险和重大群体性事件发生。跟踪落实省市金融机构支持娄底实体经济发展政银企合作对接会签约贷款，极力争取省市金融机构加大对我市的金融支持力度，特别是对具有核心竞争力、成长性好的实体企业，继续给予强有力支持，确保全市金融机构新增贷款增长12%，力争新增存贷比达到70%以上；不断创新融资手段，加快组建政府融资性担保公司，切实帮助企业解决融资难题。维护金融秩序，建立金融风险防范体系，规范民间投融资行为，依法严厉打击非法集资、恶意逃废债务、逃税避税、高利放贷和暴力收贷等行为，推进社会信用体系建设，努力构建良好的金融生态环境。

（二）进一步抓实产业转型。产业结构不优是我市经济发展遭遇困难最主要最直接的原因，转型发展是摆脱当前困境、实现可持续发展的必由之路，我们务必以“三量齐升”为目标，以“四转三化”为抓手，矢志不移推进产业转型。

加快工业转型升级。坚定不移实施“四转三化”战略，加快两化融合步伐，下大气力拉长产业链条，推动工业向集群化高端化发展。加快培育战略性新兴产业，对接长沙“中国汽车产业第六极”，重点推进薄板深加工产业园和汽车零部件基地建设，着力将娄底经开区打造为大长沙汽车产业配套协作区。加快改造传统产业，充分发挥现有重点产业、骨干企业的带动作用，有序引导钢铁、煤炭、化工等产业向中高端迈进，重点支持双峰不锈钢基地建设；做实住宅产业化钢结构基地建设前期工作，以此为契机，实现建筑材料专业化、工厂化生产，推进建筑业现代化；全力打好落后小煤矿关闭退出攻坚战，年底前全市煤矿矿点总量控制在125处以内。加快提升特色产业，打造双峰农机产业升级版，支持冷水江耐火材料、涟源煤机、新化电子陶瓷等特色产业整合提升发展。支持新化县发展文化旅游特色县域经济、申报全国特种陶瓷产业基地，支持娄星区申报省级循环经济产业园区，支持新化县、冷水江市申报全省特色县域经济重点县（市），支持涟源市加快全省农副产品加工与出口基地建设、创建全国应急产业示范基地。着力推进循环经济国家标准化试点，加快再生资源回收利用体系建设，重点加强废钢等循环利用。加快发展园区经济，落实园区“623工程”，争取用两年时间，创建6个创新创业园区，新建200万平方米标准厂房，引进300家创新创业企业；支持“飞地经济”模式，鼓励与长株潭及沿海地区合作共建。确保全市新增规模工业企业50家以上，规模工业增加值增长9%以上。

大力发展现代服务业。大力扶持商务会展、商贸物流、服务外包、文化旅游、金融保险证券、电子商务、健康养老、法律咨询等现代服务业，提高生产性服务业质量和比重。鼓励社会资本发展健康养老服务产业，全面启动众一中南医院建设，加快推进众一国际健康城建设。力争娄底农商行、冷水江农商行尽快营业，支持商业银行、民资银行等设立分支机构，争取实现县市村镇银行、农商银行“一县两行”金融发展全覆盖。正确引导房地产投资和消费，促进房地产业平稳有序健康发展。改造提升住宿餐饮、交通运输、批发零售等传统服务业。推进中小商贸流通企业服务体系建设，立足“一核二区四节点”整体布局，启动商贸流通规划编制，着力推进湘中国际物流园、湖南诚通现代物流园、电商产业园、服务外包基地等重点项目建设，加快构建区域性现代物流中心。以沪昆高铁开通为契机，支持万宝新区全力打造高铁片区商贸物流中心。实施文化旅游兴市，加快发展文化旅游产业，瞄准环长株潭“后花园”的定位，充分利用区位与资源优势，着力引进、扶持和培育文化旅游产业发展主体。加强水府庙湿地公园保护与开发，支持紫鹊界、大熊山国家森林公园、湄江国家地质公园、曾国藩故里等景区（点）提质升级，加强与大长沙旅游产业的联手合作，打造精品旅游线路，扎实推进“美丽乡村旅游扶贫工程”和旅游小镇建设，争取全年旅游经济增长18%以上。

积极发展现代农业。全面实施新增粮食产能田间工程，加强高标准农田基地建设，确保粮食播种面积410万亩以上，产量稳定在160万吨以上。推进粮油80亿产业工程，粮油工业产值增长10%以上。加快发展生态养殖，抓实标准化规模养殖小区建设，促进养殖业转型升级、提质增效。鼓励发展农机合作社、农民合作社、家庭农场、社会化农业技术服务组织等新型合作经济，有序推进土地流转，加快农业产业化经营。大力发展休闲农业和生态农业，加强农产品质量安全监管，重点推进农产品“三品一标”认证，做大做强“三黑一菌”等特色优势品牌。继续推进“菜篮子”工程建设，搞好农产品加工销售，提高产品竞争力。支持桥头河现代农业综合产业园发展，建设一批现代农业示范园区，新增规模化、标准化农产品产业基地5万亩以上，农产品加工产值增长15%以上。加快重点水源工程建设，完成小型病险水库除险加固299座，抓好全国农田水利重点县建设。加快先进适用农机推广使用，提高农业机械化水平。加强林业资源的开发利用，大力发展林产业。强化动植物防疫、森林防火、防汛、气象服务等工作，保障农业生产安全有序。

（三）进一步抓实投资增长。项目是经济发展的载体，扩大有效投资依然是拉动我市当前和今后一个时期经济发展的第一引擎，务必千方百计争取支持，筹集资金，推进项目，以项目扩投资，以投资促发展。

千方百计争取投资。用好用活用足已争取到的重大政策，把准国家投资方向和重点，加强沟通衔接，争取更多项目列入国、省规划储备笼子，确保争取上级资金占全省份额的7%以上。加强市县联动，加大政府债券和专项债券发行力度，强化财政资金引导效能。大力招商引资，深入实施“娄商返娄”战略，积极筹备第四届娄商大会，强化产业链招商、点对点招商，有针对性地举办战略性新兴产业等专题招商活动，力争全年实际利用外资、引进市外境内资金分别增长13%和10%以上。有效激活民间资本，放宽准入门槛，出台鼓励措施，在产业发展、城镇建设、基础设施等领域，优选一批规模小、周期短、见效快的项目，采用PPP等新的融资方式吸引民资投入。

准确把握投资重点。紧盯国家七大工程和省政府“三大”重点，围绕基础设施、新型工业、新型城镇、生态环保、社会民生、现代服务业六大方向，对接六个“四年行动计划”和“融城战略”，优化投资结构。全年安排重大项目700个，年度计划投资500亿元，确保100个重大项目竣工投产、200个项目开工建设、200个项目加快推进。确定市级重点项目408个，总投资1285亿元，年度投资273.9亿元，其中，工业项目87个，年度投资63.7亿元；交通项目22个，年度投资41.9亿元；城建项目88个，年度投资65.1亿元；商贸物流旅游项目61个，年度投资48.7亿元；社会事业项目67个，年度投资22.4亿元；农业农村项目62个，年度投资32.1亿元。各县市区重点项目625个，总投资1612.4亿元，年度投资367.8亿元。同时，以谋划“十三五”为引领，认真研究国家的微刺激政策，加大包装力度，强化项目谋划的深度和广度，做到上级有投资倾向，我市有项目对接，确保全年储备项目200个以上，其中产业项目100个以上。

确保项目顺利推进。继续开展“项目建设推进年”活动，进一步强化“以项目看能力、以项目论英雄、以项目评业绩”的理念，在继续坚持“五个项目组”、“四个一批”、“五个一”项目建设机制的基础上，进一步强化抓实项目的针对性和有效性，紧紧围绕文化旅游、现代物流、钢铁后续产业、健康服务业、资源循环利用产业及交通、工业地产等事关娄底发展命脉的重大项目，实行“三专”服务和调度，以钉钉子的精神推进项目建设，着力优化投资环境，构建项目审批“绿色通道”，以“保姆式服务”营造项目建设环境。依法严肃查处敲诈勒索企业的违法犯罪活动，毫不手软打击无理刁难、拦路堵门等不法行为，对“三强”行为及其背后的保护伞，对聚众闹事、寻衅滋事、干扰破坏重点项目建设的人和事，坚决做到露头就打、除恶务尽，切实解决征地拆迁难、项目审批难、施工环境差等问题。

（四）进一步抓实城乡统筹。城镇化是现代化的必由之路，城乡统筹是新型城镇化的主攻方向，务必站在为民生计、为发展计、为长远计的高度，加快推进融城战略，切实解决发展空间受限、承载能力不强的问题。

加快推进融城战略。按照《娄底市推进新型城镇化实施方案（2014－2020）》，强化规划引领，发挥区位优势，积极对接大长沙，主动融入长株潭，提高融城发展的质量和效益。进一步统一认识，加快行政区划调整步伐，力争取得实质性突破。以市县统筹、城乡一体、产城融合、节约集约、生态宜居、和谐发展为目标，大力推进交通、产业、文化、生态和观念融城，加快娄涟双、冷新经济版块建设，以娄底大道为主轴，着力打造娄双高附加值两型产业走廊、娄涟冷现代制造业走廊和中心城区国家重要的物流节点及新化大梅山重点文化旅游产业区。加快娄底大道、城市环线、站场设施等路网建设，争取启动龙琅高速公路、沪昆高速复线醴娄段建设，积极推进安邵高速、安张衡铁路建设，重点支持新化、冷水江高铁连接线建设。抓好娄邵铁路改造，确保年内通车。扎实推进县（市、区）及重点镇快速通道、重点景区旅游通道建设。建立农村公路可持续发展体系，加强农村公路建设与养护，争取冷水江军温公路改造获得上级支持。严格沿线小城镇、主要干道两厢和重点乡镇土地的用途管制，优化沿线产业的规划布局，着力发展一批产业特色镇、物流专业镇、生态文化旅游休闲镇。启动户籍制度改革，吸纳农业转移人口落户城镇。

增强综合承载能力。按照“南扩北延、东优西联”的思路，推进中心城区的提质改造扩容。加快基础设施建设，统筹电力、通信、供水、治污、垃圾处理、燃气工程及地下管网建设，力争年底中心城区大部分居民用上天然气。抓好水府庙水源保护、新化二水厂建设，确保城镇供水安全。加强防洪设施建设，完善城市排水与外洪内涝防治体系。加强小区物业管理，优化社区生活设施布局，完善服务网络。加快配建城市停车场、强化交通秩序管理，缓解停车难问题。

改善城乡人居环境。更加注重城市管理转型提质，构建城市管理长效机制。全面加强城管行政执法，继续加强店外经营、门店垃圾、渣土、广告等专项整治。加快环卫、绿化管养等市场化步伐。推进数字城管建设，加强精细化、网格化管理，营造“全民城管”氛围。巩固提升创卫成果，持续开展城乡环境整治与建设，推进背街小巷改造、城区美化、亮化、绿化等工程，完善城镇污水处理及再生利用设施，推进雨污分流改造、污泥无害化处置和水泥窑协同处置生活垃圾，提高城乡污水处理和垃圾无害化处理能力。推进涟钢周边、关闭矿山环境综合治理及锡矿山地区环境整治与生态修复，抓好地质环境治理与灾害防治工作。推进大气污染防治，对电力、钢铁等重点行业实行二氧化硫和氮氧化物排放总量控制，开展机动车尾气氮氧化物治理，空气环境质量及时向社会公布。加快推进新农村建设，加强农村建设的规划管控，按照“四清、三基、两统、一化”要求，建立镇区整治及农村长效保洁机制，推进垃圾分类减量无害化处理。促进农村污染减排，抓好规模化畜禽养殖污染防治。推行最严格的水资源管理制度，推进湘江保护一号重点工程，重点开展“四水三库”的保护与治理，争取把资江治理纳入国家笼子，实施水环境保护与治理“三年行动计划”，确保四水干流水质达到Ⅲ类以上标准，城镇集中式饮用水源保护区水质达到Ⅱ类以上标准。

（五）进一步抓实改革创新。适应新常态，尤其需要通过改革创新来破解难题、激发活力、催生动力、释放红利，我们务必以全面深化改革为契机，不断开拓创新，强推开放合作，着力推进发展。

全面深化改革。加快投融资体制改革，推进PPP等混合所有制投资模式，激发投资活力。理顺园区管理体制，逐步解决经开区放权、万宝新区体制机制理顺的问题，力争园区所有审批事项在区内一站式审批办结。巩固“两集中两到位”成效，推动权力清单、责任清单和市场准入负面清单公布运行，做到该精简的精简，该下放的下放。同时，厘清乡镇权力清单、责任清单。围绕建立现代财政制度，着力构建科学规范的收入管理机制、完善透明的预算管理机制、运行高效的绩效管理机制。加强政府性债务化解与监管、财政专项资金和税费征收成本管理，着重解决管理粗放、分配不公、效益不高等问题。加强机构编制管理，稳妥推进行政机构改革和事业单位分类改革。按照统一部署，全面推行公车改革。完善审计制度，加强审计结果运用，保障审计机关依法独立行使审计监督权。深化文化体制改革，鼓励组建文化产业集团。加快农村综合改革，稳妥推进农村土地制度改革试点。深化国企改革，推进政企、政资分开。

突出创新驱动。强化企业创新主体地位，激发企业自主创新意识。深化产学研合作，对创新企业给予真金白银的支持。重点是立足传统优势产业和特种陶瓷、装备制造等特色产业，加强核心关键技术攻关，把创新落实到新的增长点上。加快建设一批研发中心、孵化基地、实验室，夯实创新平台，争取科技重点项目和重大专项支持。以市科技综合服务平台为载体，促进科技资源整合共享与高效利用。实施知识产权战略，加大知识产权保护力度。力争新增高新技术企业90家，高新技术产品产值达到720亿元。

促进开放合作。积极对接大长沙的优势产业项目、产业链、产业园，探索建设“飞地园区”，主动融入大长沙的产业集群。紧扣提质扩量，进一步调优外贸结构，扩大外贸总量，引进外向型企业发展加工贸易，发挥出口拉动作用。建设外贸综合服务平台，加快铁路口岸建设，完善市场拓展与关检服务，提高货物的流转速度和通关效率。紧扣“走出去”战略，逐步扩大境外投资和劳务输出规模，提升经济外向度。

（六）进一步抓实民生改善。民生是发展之本，也是我们党执政之本、政府行政之本，越是困难的时候越是要注重改善民生，我们务必以全面小康建设为总揽，在推进发展中，更多地考虑群众的利益和诉求，有计划、有步骤地解决好民生问题。

努力增加群众收入。落实各项支农惠农政策，促进农民增收。积极对接国家武陵山片区发展规划，实施精准扶贫，加快落后地区的发展，减少贫困人口9万人。做好移民安置和后期扶持工作，争取涟源市纳入全国水库移民避险解困第二批试点，全市移民人均纯收入增长13%以上。继续实施积极的就业政策，优化就业创业环境，抓好市公益性创新创业孵化基地建设，加大高校毕业生、下岗失业人员、返乡农民工、退役士兵、残疾人等困难人群帮扶力度，城镇登记失业率控制在4.3%以内，新增城镇就业3.7万人以上，新增转移农村劳动力5万人以上，完善最低工资和农民工工资保障制度。大力开展治理拖欠农民工工资问题专项行动，从严惩处不良企业和业主，竭尽全力让广大农民工拿到自己应得的劳动报酬。

完善社会保障体系。集中财力办实事，健全覆盖城乡的社会保障体系，建立统一的城乡居民养老保险制度，全面落实医疗、工伤、生育、失业保险制度。强化社会救助体系建设，深化城乡低保“阳光行动”,加大重特大疾病医疗救助和临时救助力度，启动城乡居民大病保险、重度残疾人补助制度，给特殊困难群众以特别关怀。加快构建覆盖城乡的社会养老服务体系，新增养老床位1500张。加快“两房两棚”建设，改善困难群众居住环境。新建保障性住房26606套，改造农村危房6000户。解决农村24.5万人饮水不安全问题。关心关爱留守儿童，支持慈善事业发展，竭尽全力让每一个身处困境的人都能得到关爱和温暖。

全面发展社会事业。优先发展教育事业，大力推进学前教育普惠性发展、义务教育均衡发展、普高教育特色发展、职业教育创新发展，鼓励发展民办教育。推进农村学校教师周转宿舍建设，扎实开展标准化学校试点，确保教育专项资金专款专用，努力改善薄弱学校、边远学校和特殊教育学校办学条件。继续化解大班额，年内新增学位9300个。加强教师队伍建设，提升教育质量。加快公共文化标准化、均等化布局步伐，推进县市文化馆、博物馆建设，完成“村村通”、“户户通”建设任务。全面推进县级公立医院综合改革，推动分级诊疗，引导群众有序就医。完善医疗纠纷第三方调解机制，落实医疗机构治安管理责任，严厉打击涉医违法犯罪，构建和谐医患关系。稳步推进人口计生工作，坚持计生工作“一票否决制”不动摇，确保人口自然增长率控制在7.5‰以内。健全食品药品安全监管体制，推进食品药品检验实验室建设，抓好生猪屠宰自动流水线建设，确保食品药品安全。加强公共体育设施建设，扎实推动全民健身，促进体育消费。进一步加强统计基层基础，扎实做好全面小康监测工作。加快市档案馆建设。

推进多层次多领域依法治理。依法抓实抓严生产安全，全面落实安全生产责任制，抓实煤矿、非煤矿山、交通、危化物品、建筑施工、粉尘防爆、地质灾害等重点领域生产安全，狠抓电梯等特种设备、消防、校车等公共安全，全面推进公路治超，有效遏制各类安全事故发生。深化平安娄底建设，严厉打击违法犯罪活动，加大禁毒工作力度，完善立体化治安防控体系，推动“天网工程”向乡镇延伸。强化社会稳定风险评估,完善矛盾纠纷化解体系，畅通诉求表达渠道,依法打击缠访、闹访，积极防范和稳妥处置群体性事件。加强应急管理与突发事件预警系统建设，提高公共安全和防灾救灾减灾能力。加强网络舆情管理与引导，规范网络行为。深化社会主义核心价值观教育，发挥乡规民约在社会治理中的积极作用，提高市民文明素质。加强普法教育与法律援助，推进基层治理法治化。关注基层、服务基层，加强社区、乡村基层组织建设，健全社区网格治理体系，探索推进农村网格化治理。激发社会组织活力，形成多元主体、协同共治格局。努力提高基层干部待遇，让他们更好体现价值、扎根基层。

加强国防和后备力量建设。大力推进与国家发展战略相顺应、与娄底经济社会发展相互动、与履行职能使命相对称的国防动员和后备力量建设，增强全民国防教育时效，巩固深化双拥活动成果，推动军民融合深度发展，着力提高应急应战准备质效，不断增强遂行多样化任务能力，为巩固国防和政权、确保社会和谐稳定提供坚强可靠的力量支撑。

科学编制“十三五”规划，做到谋长远、早部署。在发展目标上，准确把握新常态，确保提出的重大项目、重大政策、重大工作目标可行、措施可靠、效果可期。在具体措施上，加强沟通汇报争取，力争更多的重点项目列入国家、省规划盘子，确保争取项目资金超过市州平均份额。在工作要求上，结合改革发展需要，积极谋划新思路、新举措，确保开好局、起好步。

三、加强政府自身建设

面对新形势、新任务、新常态，我们要始终坚持以人为本、执政为民，把群众需求作为根本导向，把依法行政作为基本标尺，把优化作风作为重要保障，努力建设为民务实清廉法治政府。

更新观念，切实增强创造力。进一步解放思想，坚决消除封闭抵制、固步自封的传统思想，切实强化着眼于整体发展的大局意识、着眼于长远发展的包容胸怀，不争论、不折腾、不懈怠，既敢于走出去，也乐于引进来，以思想的大解放推动改革的大突破。坚决消除狭隘固化的地域观念和个人利益、部门利益至上的自私心态，大力推进强强联手、协作发展、互利互惠，以观念的大更新推动市域的大开放。坚决消除躺在资源上睡大觉的惰性思维和只能依赖资源发展产业、必须依赖资源发展产业、依赖资源好发展产业的惯性思维，以从零开始的态度、无中生有的智慧，引进新兴产业特别是战略性新兴产业，以思维的大拓展推动经济的大发展。

主动作为，切实增强执行力。越是面对困难和挑战，越要主动作为、积极有为。要敢于负责，充分发挥我们娄底人“敢为天下先”的勇气和胆识，身在其位、勇谋其政、敢尽其责，把该抓的抓到位，该管的管具体，把困难想充分，把工作做扎实，踏踏实实谋事业，一心一意促发展。要勤于负责，充分发扬我们娄底人“霸得蛮、吃得苦”的精神，以一抓到底的勇气扭住项目不放，以不出成效不撒手的决心促进经济发展。始终坚持有令必行，立说立行，雷厉风行，始终保持一种动真格的冲劲、求突破的钻劲、见实效的拼劲、不懈怠的韧劲，定了就干，干就干好，干就干成。要善于负责，充分发挥我们娄底人极具智慧、勤奋干练的特质，弘扬钉钉子精神，定任务、定人员、定要求、定时间，一级带着一级干、一级做给一级看、一级督促一级办，做到人人肩上有担子，件件事情有着落。

依法行政，切实增强公信力。着力建设法治娄底，将法治思维贯彻到改革发展各方面、贯穿于政府工作全过程，不断提高运用法治思维和法治方式解决问题、推动工作的能力，切实做到法无授权不可为、法定职责必须为。着力抓好市县政府机构改革，进一步转变职能，精减行政审批事项，优化行政审批程序，努力提升行政效能。自觉接受人大法律监督与工作监督、政协民主监督、社会舆论监督，牢固树立有权必有责、用权受监督、违法严追究的观念，坚决改变规避法律、以权压法的错误思维，坚决惩处徇私枉法、野蛮执法等违法违纪事件，扎实推进决策公开、执行公开、信息公开、结果公开，让群众真正感受到法治政府建设带来的新变化。

正风肃纪，切实增强约束力。严格遵循中央“八项规定”和省委“九项规定”，守纪律讲规矩，坚决做到令行禁止、政令畅通，切实解决决策不民主、程序不规范、执行不严格等问题。巩固党的群众路线教育实践活动成果，横下一条心坚决纠正“四风”，强力整治慵、懒、散，拖、推、慢等不良习气，严肃查处不作为、慢作为、乱作为、办事不公、吃拿卡要等行为。始终坚持以廉服众，认真履行“一岗双责”，强化“两个责任”，注重加强制度建设，切实增强抵御诱惑、守住底线、廉洁自律的定力。保持反腐败高压态势，做到无禁区、全覆盖、零容忍，坚决查处腐败案件，以清正廉洁的形象取信于民。

各位代表，新常态承载新使命，新目标更须新作为。让我们在市委的坚强领导下，振奋精神、坚定信心，敢于担当、善做善成，为全面建成小康娄底而努力奋斗！
